# Hemoglobin levels in red blood cells and risk of colorectal cancer: A causal investigation based on Mendelian randomization

**DOI:** 10.1097/MD.0000000000040562

**Published:** 2024-11-29

**Authors:** Jin Shen, Xiuyuan Qin, Xiang Zeng, Hanyu Xiao, Suhe Lai

**Affiliations:** a Bishan Hospital of Chongqing, China; b Bishan Hospital of Chongqing Medical University, China; c Chongqing Medical University, China.

**Keywords:** colorectal cancer, mean corpuscular hemoglobin, Mendelian randomization

## Abstract

Mean corpuscular hemoglobin (MCH) is a critical parameter in red blood cells, associated with various diseases. While studies suggest a potential link between MCH levels and colorectal cancer (CRC), observational studies are insufficient to establish causality directly. This study utilized a 2-sample Mendelian randomization (MR) approach to investigate the genetic causal relationship between MCH and colorectal cancer (CRC). Genome-wide association study (GWAS) summary data for both MCH and CRC were sourced from relevant databases. MR analyses were performed using methods including inverse variance weighted (IVW), MR Egger, weighted median, simple mode, and weighted mode. Cochrane’s *Q* test was applied to assess heterogeneity in the MR findings. Horizontal pleiotropy was evaluated using the MR-Egger intercept test and the MR Pleiotropy RESidual Sum and Outlier (MR-PRESSO) test. Additionally, a leave-one-out analysis was conducted to assess the robustness of this association. The IVW method demonstrated that MCH is an independent risk factor for colorectal cancer (*P* = .013). Horizontal pleiotropy is unlikely to influence the causal relationship (*P* > .05), and there was no evidence of heterogeneity among the genetic variants (*P* > .05). Lastly, the leave-one-out test confirmed the stability and robustness of the association. All participants in the GWAS were derived from a specific population. Due to limitations inherent to the database, the Mendelian Randomization (MR) analysis was unable to incorporate stratified analyses by country, ethnicity, or age group.

## 1. Introduction

Colorectal cancer (CRC) represents over 10% of all global cancer diagnoses, ranking as the third most prevalent cancer and the second leading cause of cancer-related mortality. It is estimated that annually there are 1.9 million new cases and 935,000 deaths.^[1,2]^ Notably, the incidence of CRC is on the rise, particularly among younger demographics (under 50 years).^[3]^ Given these challenges and the projection that 50% of CRC cases are preventable,^[4]^ it is imperative to identify novel risk factors and develop corresponding prevention and treatment strategies to alleviate future healthcare burdens.

Anemia frequently occurs among patients with CRC, with research indicating that diminished hemoglobin levels are linked to systemic inflammation and increased tumor aggressiveness. Specifically, reduced mean corpuscular hemoglobin (MCH) correlates with a poorer prognosis in CRC patients and has been recognized as an independent prognostic factor for decreased survival rates.^[5,6]^ These hematological parameters are instrumental in predicting disease recurrence and overall survival rates.^[7,8]^

In this study, we aim to investigate the relationship between MCH and the incidence of CRC. To achieve this, we employed the most comprehensive genome-wide association study (GWAS) available, conducting Mendelian randomization (MR) analyses to explore the associations between MCH and CRC. These analyses allow us to compare genetic proxies with observational estimates, providing a thorough examination of the potential link between MCH levels and CRC development.

## 2. Data sources

**Figure 1.**
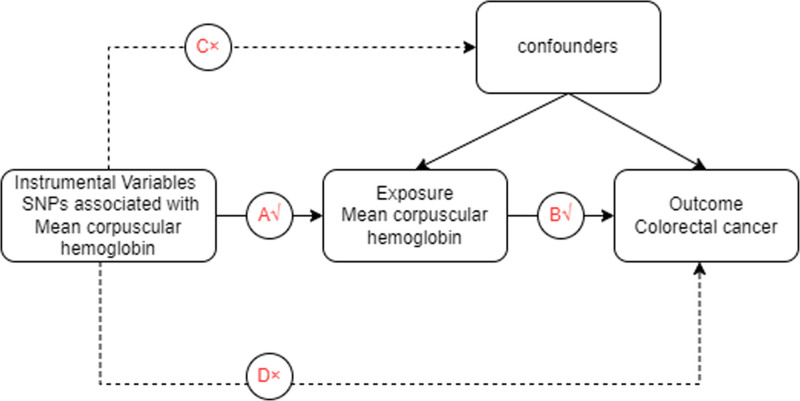
Schematic diagram of MR.

By querying the open EBI database of the Comprehensive Epidemiology Unit with the search term ‘mean corpuscular hemoglobin’ we obtained the dataset ebi-a-GCST004630. This dataset comprises single-nucleotide polymorphism (SNP) data from 172,332 samples, totaling 29,166,133 SNPs, each documented with mean corpuscular hemoglobin levels. For GWAS data pertaining to colorectal cancer (CRC), we utilized dataset ebi-a-GCST90018808, which includes 470,002 samples and 24,182,361 SNPs. In this study, CRC is designated as the outcome variable, while mean corpuscular hemoglobin levels are examined as the exposure variable. Figure 1 provides an overview of the study design.

## 3. Data preprocessing

MR studies are based on 3 fundamental principles: a strong and statistically significant correlation between instrumental variables (IVs) and the focal exposure; independence of IVs from potential confounders; IVs exclusively affecting the outcome through the specified exposure pathway without other alternative routes. The exposure variable was assessed, and IVs were screened using the “extract_instruments” function in the TwoSampleMR package (*P* < 5 × 10^−8^). The “clump” setting was set to TRUE to conduct linkage disequilibrium analysis (*R*^2^ = 0.001 and kb = 10,000). An *R*^2^ value of 0 indicates complete linkage equilibrium between 2 SNPs, meaning their allocation is entirely random. IVs significantly associated with the exposure variable were selected. Outcome variables were extracted using the “extract_outcome_data” function in the R package. IVs derived from the exposure variable were merged with the screening criteria, with the proxy setting set to TRUE. Additionally, weak IVs were omitted based on the *F* statistic of each SNP. The formula *F* = *R*^2^/ (1 − *R*^2^) × (N − *K*) / (*K* − 1), where *R*^2^ represents the cumulative explained variance of selected IVs concerning the exposure factor. N denotes the sample size of the GWAS, and K indicates the number of SNPs screened for the exposure. An *F* statistic ≥ 10 indicates no weak instrument bias, thus highlighting the strong predictive power of IVs on the outcome.

## 4. MR analysis

In the TwoSampleMR package, the “harmonize_data” function was used to standardize effect estimates. Key MR techniques included MR-Egger, weighted median, and inverse variance weighted (IVW) methods, employing both multiplicative random effects and fixed effects. The IVW method was emphasized in the primary analysis, which requires SNPs to fully comply with the 3 core MR principles to obtain accurate causal estimates. MR-Egger adds an additional intercept term primarily to determine the presence of horizontal pleiotropy. The weighted median method uses the majority of SNPs (most genetic variants) to assess the existence of a causal relationship. If the IVW causal estimate of a given variable lacks additional heterogeneity, the results from both random effects and fixed effects will be the same, resulting in no loss of precision. When the IVW method produces noteworthy results (*P* < .05), it can be considered positive even if other methods’ results are not significant, provided the directionality of β values between methods is consistent, and no pleiotropy or heterogeneity exists. Subsequently, study results were visually presented using scatter plots, forest plots, and funnel plots. In scatter plots, the IVW method remains the main focus, with very small intercepts indicating minimal confounder effects and no impact on result reliability. A positive slope of the line indicates a risk factor, while a negative slope indicates a protective factor. Forest plots aim to evaluate the diagnostic efficacy of each SNP locus in predicting outcome diagnosis based on the exposure variable, with solid dots on the left indicating a decrease and those on the right indicating an increase, focusing on the IVW position. Funnel plots were constructed to assess randomness; symmetrical distribution of IVs on either side of the IVW line indicates that MR complies with Mendel’s second law: random assortment.

## 5. Sensitivity analysis

To assess the robustness of MR analysis results, a sensitivity analysis was conducted. First, heterogeneity detection was performed. A Cochran’s *Q* test *P* value >.05 indicated a lack of heterogeneity. Subsequently, a horizontal pleiotropy test was conducted, with *P* values exceeding .05 indicating no horizontal pleiotropy effect, suggesting no confounding factors in the study. Finally, a leave-one-out (LOO) analysis was conducted by iteratively removing each SNP. The consistency of the effects of the remaining SNPs on the outcome variable confirmed the reliability of the MR analysis results.

## 6. Results

### 6.1. Causal relationship between MCH and CRC

**Figure 2.**
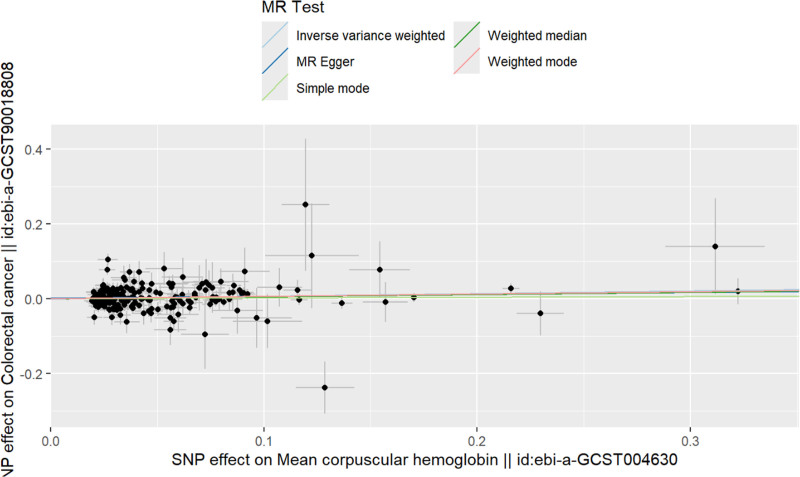
The scatter plot of MR analysis of MCH and CRC. CRC = colorectal cancer, MCH = mean corpuscular hemoglobin, MR = Mendelian randomization.

After thorough screening, 205 SNPs were identified as instrumental variables (IVs). Subsequent MR analysis evaluated the effect of mean corpuscular hemoglobin (MCH) on CRC within the UK population, considering MCH as the exposure and CRC as the outcome. Results from 4 distinct MR methods uniformly indicated a positive causal relationship between MCH and CRC. The lowest *F* statistic recorded was 174.781, validating the reliability of these findings and establishing MCH as a significant risk factor (IVW model: odds ratio = 1.074, *P* = .013; Table 1). To further elucidate this relationship, scatter plots of the SNPs were generated, revealing a positive linear trend; higher MCH levels correlate with an increased probability of developing CRC (Fig. 2).

**Table 1:** The MR results by 5 methods.

Exposure	Outcome	Method	SNP (n)	OR	OR 95% CI		*P* value
MCH	CRC	MR-Egger	205	1.054	0.955	1.163	.295
MCH	CRC	Weighted median	205	1.061	0.987	1.140	.104
MCH	CRC	IVW	205	1.074	1.015	1.138	.013
MCH	CRC	Simple mode	205	1.018	0.876	1.184	.809
MCH	CRC	Weighted mode	205	1.064	0.996	1.136	.063

CRC = colorectal cancer, IVW = inverse variance weighted, lci95 = lower 95% confidence interval, MCH = mean corpuscular hemoglobin, MR = Mendelian randomization, OR = odds ratio, SNP = single nucleotide polymorphisms, uci95 = upper 95% confidence interval.

**Figure 3.**
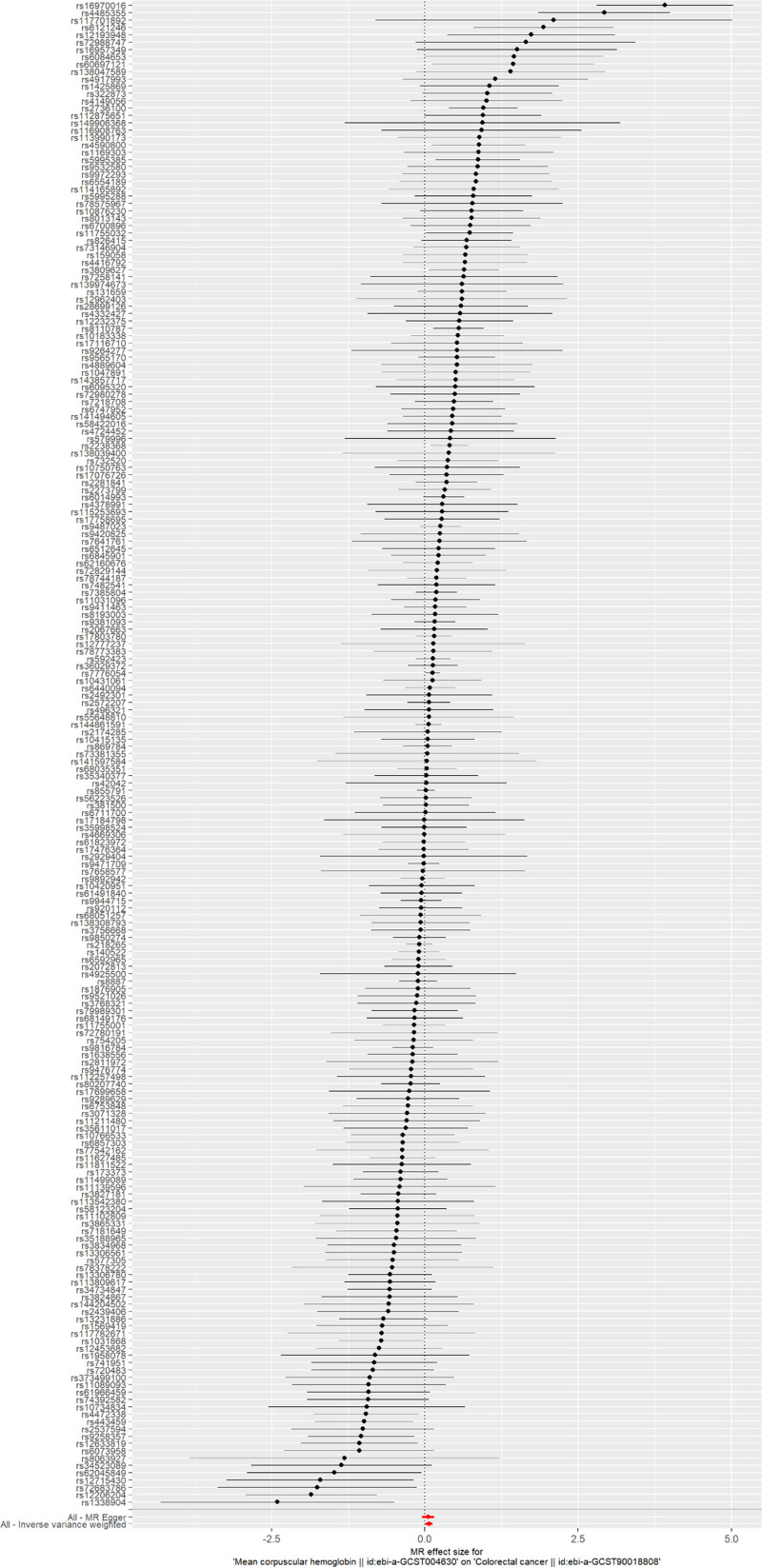
The forest plot of MR analysis of MCH and CRC. CRC = colorectal cancer, MCH = mean corpuscular hemoglobin, MR = Mendelian randomization.

**Figure 4.**
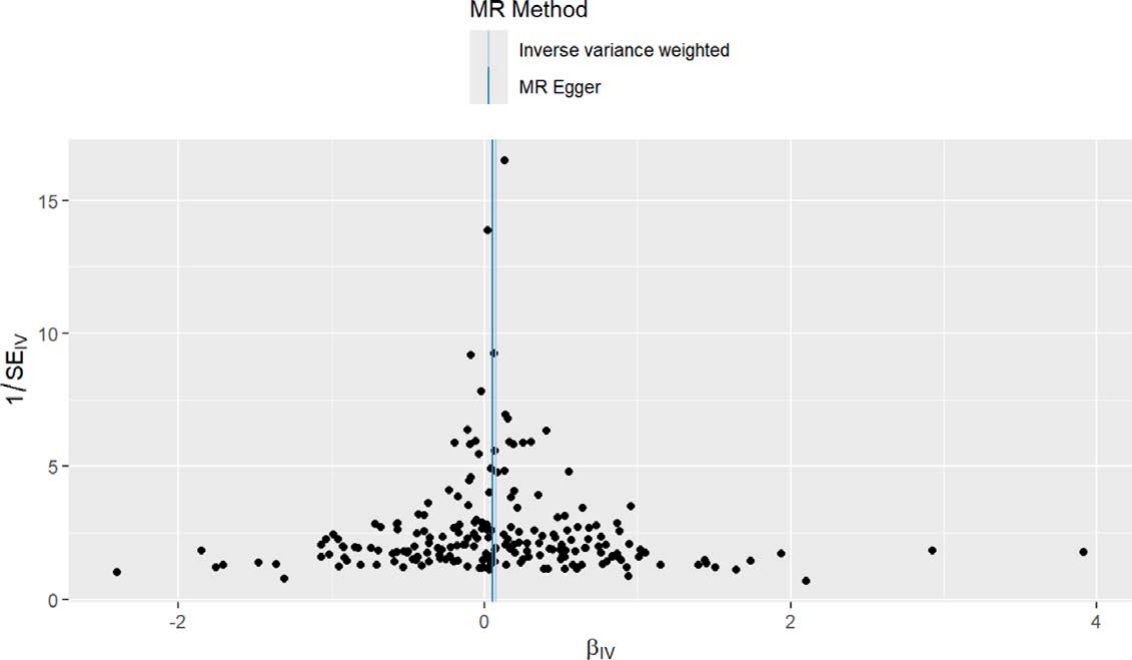
The funnel plot of MR analysis of MCH and CRC. CRC = colorectal cancer, MCH = mean corpuscular hemoglobin, MR = Mendelian randomization.

Forest plots were constructed to assess the predictive efficacy of each SNP locus in relation to the exposure factor and the outcome. In these plots, solid dots positioned to the left signify a lower risk, whereas those on the right indicate a higher risk. The forest plots uniformly positioned the solid dots on the right, suggesting that increased exposure factors raise the risk of disease, as demonstrated by the IVW method (Fig. 3). Additionally, the randomness of the instrumental variables (IVs) was verified and visualized through funnel plots, which displayed a symmetrical distribution of IVs on either side of the IVW line. This symmetry confirms that the MR analysis adheres to the principle of random assortment (Fig. 4).

### 6.2. Sensitivity analysis

To ensure the robustness of our conclusions, comprehensive sensitivity analyses were conducted. Initially, all Q_pval values from heterogeneity tests exceeded 0.05, indicating an absence of heterogeneity among the samples and reinforcing the primary use of the IVW (fixed effects) method in the MR analysis. Subsequent tests, including MR-Egger and MR-PRESSO regression, were employed to assess potential horizontal pleiotropy effects from the genetic IVs. These tests uniformly showed no evidence of such effects between MCH and CRC (*P* > .05).

**Figure 5.**
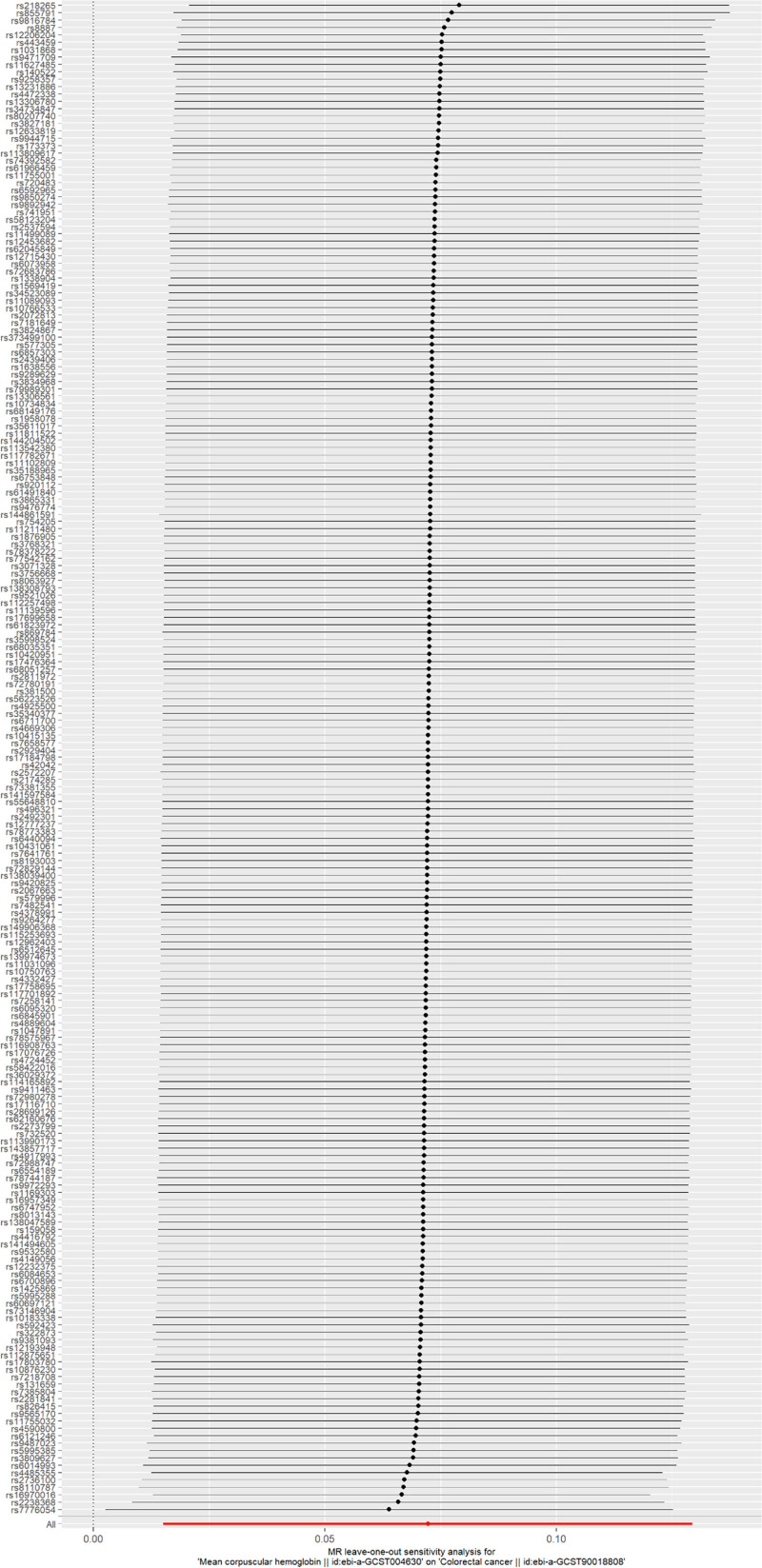
The leave-one-out sensitivity plot of MR analysis of MCH and CRC. CRC = colorectal cancer, MCH = mean corpuscular hemoglobin, MR = Mendelian randomization.

Furthermore, a leave-one-out (LOO) analysis was conducted to verify the reliability of the results. This analysis involved iteratively excluding 1 SNP at a time and performing MR with the remaining SNPs to evaluate the influence of a specific SNP on the outcome. The primary objective of the LOO analysis was to determine whether the trajectory connecting the black dots in the plot remained smooth and free of noticeable outliers. The results of the LOO analysis revealed no significant outliers, thereby affirming the reliability of the findings (Fig. 5).

## 7. Discussion

This study explored the causal relationship between MCH and CRC via 2-sample Mendelian randomization (MR) analysis using extensive GWAS data. The results demonstrate that elevated MCH levels correlate with an increased risk of CRC. These findings suggest that interventions aimed at modulating hemoglobin levels in red blood cells could potentially reduce the incidence of CRC. Consequently, this offers vital biological insights for developing preventive strategies, particularly for high-risk groups, potentially diminishing disease burden and enhancing clinical outcomes.

Increasing evidence indicates that MCH and other hematological parameters are associated with both the risk and prognosis of CRC. MCH quantifies the average amount of hemoglobin per red blood cell, a critical factor for tissue oxygen transport. Abnormal MCH values may indicate disruptions in tissue oxygenation, potentially contributing to hypoxia: a condition that fosters tumor growth and invasion. Hypoxic environments may activate several signaling pathways, including hypoxia-inducible factor 1α (HIF-1α), which supports tumor proliferation. Research has demonstrated that total hemoglobin concentration (THC) increases, while oxygen saturation (StO₂) decreases in CRC tissues as tumors evolve from benign to malignant states, signifying a shift toward hypoxia within the tumor microenvironment.^[9]^ Key regulatory mechanisms involving the mTOR pathway and PP2A phosphatase affect PHD2 phosphorylation, thereby controlling HIF1α levels essential for cellular survival in hypoxic conditions. These hypoxia-induced alterations result in HIF1α stabilization, promoting both autophagy and survival in CRC cells.^[10]^ The activation of hypoxia-inducible factors (HIFs) in response to oxygen deprivation orchestrates the regulation of genes essential for oxygen supply and energy metabolism, vital for sustaining tumor growth under low oxygen conditions.^[11]^ Furthermore, hypoxia-driven microRNAs, such as miR-210, enhance CRC self-renewal and tumorigenesis by adjusting metabolic pathways and increasing lactate production, thus facilitating the survival and expansion of tumor-initiating cells under hypoxic conditions.^[12]^ Additionally, hemoglobin may boost CRC cell proliferation by generating reactive oxygen species (ROS), underscoring a direct connection between hemoglobin levels and tumor progression.^[13]^ CRC cells adapt to hypoxic states by modifying metabolic pathways, increasing glycolysis, and upregulating enzymes linked to anaerobic metabolism, which supports ongoing growth and resistance to treatments.^[14]^

Hypoxia may indirectly influence tumor progression by enhancing systemic or localized inflammatory responses, which are acknowledged as catalysts for cancer development, particularly in CRC. Abnormal MCH levels are often linked to chronic inflammatory states that may encourage CRC onset and progression. Anemia, prevalent among CRC patients, correlates with systemic inflammation. Specifically, the type of anemia (predominantly normocytic) is associated with higher Glasgow Prognostic Score (mGPS), elevated C-reactive protein (CRP), and interleukin-8 (IL-8) levels, underscoring a significant link between anemia and systemic inflammation.^[5,15]^ Chronic inflammation is a critical risk factor for CRC. Interactions between immune cells, stromal cells in the tumor microenvironment, and the gut microbiome may suppress or circumvent immune responses, thereby facilitating tumor growth and progression.^[16]^ Moreover, host inflammation in CRC substantially impacts red blood cell iron status, with studies indicating significant correlations between changes in CRP and albumin levels and alterations in red blood cell parameters like hemoglobin (Hb), mean corpuscular volume (MCV), and MCH, reflecting the effect of systemic inflammation on iron metabolism.^[17]^ Chronic inflammation can also drive tumor formation through epigenetic alterations. For instance, DNA methylation changes triggered by chronic inflammation may deactivate genes that are crucial for intestinal homeostasis and damage response, a phenomenon particularly pertinent in CRC.^[15,18]^ Furthermore, hematological inflammatory markers such as the neutrophil-to-lymphocyte ratio (NLR), platelet-to-lymphocyte ratio (PLR), and systemic immune-inflammation index (SII) are closely associated with CRC diagnosis and prognosis. Elevated levels of these markers correlate with tumor location, TNM stage, and systemic inflammation.^[19]^ Additionally, alterations in hemoglobin are significantly linked to systemic inflammatory responses in CRC, with low hemoglobin levels (<10 g%) associated with increased anaerobic metabolism and high LDH5 expression, factors closely related to tumor invasion and metastasis.^[14]^

Changes in MCH levels may indicate abnormalities in iron metabolism, which is essential for cell growth and division.^[6,20]^ Imbalances in iron metabolism, either deficiency or overload, have been linked to an increased risk of various cancers. Specifically, iron overload may escalate cancer risk by generating free radicals, elevating oxidative stress, and thereby increasing DNA damage and mutations. Iron plays a crucial role in several biochemical reactions and physiological processes, including the normal cell cycle, mitochondrial function, nucleotide metabolism, and immune responses. Dietary iron, particularly heme iron, is identified as a significant risk factor for CRC. Furthermore, CRC patients frequently exhibit abnormal iron absorption, storage, utilization, and export, highlighting iron’s involvement in CRC development and progression.^[20]^ Research has shown that iron concentrations in CRC tissues are significantly higher than in polyp tissues, while serum iron levels in CRC patients are notably lower than in those with polyps and controls, suggesting an association between iron accumulation in tumor tissues and reduced serum iron.^[21]^ Epidemiological and experimental studies have proposed that heme iron contributes to CRC by facilitating the formation of carcinogenic N-nitroso compounds and producing cytotoxic and genotoxic aldehydes through lipid peroxidation.^[22,23]^ Iron deficiency anemia is prevalent in CRC patients, with studies indicating that female gender, right-sided colon tumors, and larger tumors are risk factors for this condition. Patients with low serum ferritin levels are more prone to developing CRC, suggesting a link between iron deficiency and CRC onset.^[24,25]^ Hepcidin, often abnormally expressed in CRC tumor epithelium, may exacerbate tumor parameters by enhancing intracellular iron accumulation through the suppression of the iron export protein ferroportin. Iron chelators have been shown to reduce mitochondrial function, alter nucleotide synthesis, and inhibit tumor growth, underlining iron’s pivotal role in CRC cell metabolism.^[26]^ Research has documented significant alterations in iron metabolism-related molecules such as ferritin, transferrin, and serum iron in CRC patients, potentially influencing tumor occurrence and progression.^[17]^

This study boasts several strengths. Primarily, to the best of our knowledge, this is the inaugural research utilizing large-scale GWAS to examine the causal relationship between MCH and CRC. The 2-sample MR approach mitigates several limitations of observational studies, including reverse causality, confounding factors, and various biases. Secondly, all instrumental variables (IVs) employed in the MR analysis underwent rigorous screening, with the lowest *F* statistic recorded at 174.781, thus bolstering the accuracy of our results. Lastly, we applied multiple methods to assess sensitivity, horizontal pleiotropy, and heterogeneity. Collectively, these tests affirm the stability and robustness of the association between MCH and CRC.

Nevertheless, this study has notable limitations. Firstly, all participants in the GWAS were of European descent; thus, it is uncertain whether our findings can be extrapolated to other populations and regions. Secondly, although we utilized MR intercept and MR-PRESSO global tests to identify and adjust for pleiotropy in genetic variants, residual confounding factors such as education level, personality, and nutrition could still influence the relationship between the exposure and outcome, potentially skewing our results. Thirdly, since our MR analysis is dependent on data from underlying GWAS meta-analyses, we were unable to conduct stratified analyses by country, ethnicity, or age group. Consequently, the effects of MCH observed in this study may not be applicable to populations with distinct characteristics, such as different races and age groups.

## 8. Conclusion

We have established a causal relationship between MCH and CRC, suggesting potential involvement of shared biomarkers or physiological pathways. Our findings provide new insights into the genetic and biological mechanisms that underpin the role of MCH in the development of CRC.

## Acknowledgments

The authors sincerely thank the researchers and participants of the original GWASs for the collection and management of the large-scale data resources.

## Author contributions

**Conceptualization:** Jin Shen, Xiuyuan Qin, Xiang Zeng.

**Data curation:** Jin Shen, Xiuyuan Qin, Xiang Zeng, Hanyu Xiao.

**Formal analysis:** Jin Shen, Xiuyuan Qin.

**Funding acquisition:** Xiuyuan Qin, Suhe Lai.

**Investigation:** Xiang Zeng, Suhe Lai.

**Methodology:** Jin Shen, Xiang Zeng, Hanyu Xiao.

**Project administration:** Xiang Zeng.

**Resources:** Jin Shen, Xiang Zeng, Hanyu Xiao, Suhe Lai.

**Software:** Hanyu Xiao.

**Supervision:** Suhe Lai.

**Validation:** Hanyu Xiao, Suhe Lai.

**Visualization:** Suhe Lai.

**Writing – original draft:** Jin Shen, Xiuyuan Qin, Xiang Zeng, Hanyu Xiao, Suhe Lai.

**Writing – review & editing:** Jin Shen, Xiuyuan Qin, Hanyu Xiao, Suhe Lai.
